# Causal relationship between PCOS and related sex hormones with oral inflammatory diseases: a bidirectional Mendelian randomization study

**DOI:** 10.3389/fendo.2023.1282056

**Published:** 2024-01-09

**Authors:** Qiusi Min, Yi Chen, Hongling Geng, Qian Gao, Xueying Zhang, Min Xu

**Affiliations:** ^1^ Guangzhou University of Chinese Medicine, Guangzhou, Guangdong, China; ^2^ Department of Gynecology, Guangdong Provincial Hospital of Chinese Medicine, Guangzhou, Guangdong, China

**Keywords:** Mendelian randomization, PCOS, mouth ulcers, painful gums, periodontitis, SNP, AMH

## Abstract

**Background:**

Observational studies have identified a strong association between polycystic ovary syndrome (PCOS) and hormone levels related to oral inflammatory diseases. To better understand the relationship between them, we conducted an analysis using a two-sample Mendelian randomization (MR) approach.

**Methods:**

We gathered summary statistical data from previously published genome-wide association studies (GWAS) on PCOS and three sex hormones (AMH, Estradiol, LH) along with four oral inflammatory diseases (painful gums, loose teeth, mouth ulcers, and toothache). We selected single nucleotide polymorphisms (SNPs) as instrumental variables and employed four types of MR analysis methods to evaluate causal relationships between exposure and outcome. Finally, the robustness of our results was further validated through sensitivity tests and reverse MR.

**Results:**

We observed that PCOS could increase the risk of mouth ulcers (*OR_IVW_* = 1.0013, *95%CI:* 1.0001-1.0025, *P_IVW_* = 0.0278), painful gums (*OR_IVW_* = 1.0015, 95%CI:1.0003-1.0027, *P_IVW_* = 0.0163), and loose teeth (*OR_IVW_* = 1.0014, *95%CI:* 1.0001-1.0027, *P_IVW_* = 0.0328). Moreover, LH was also found to increase the risk of mouth ulcers (*OR_IVW_* = 1.0031, *95%CI:* 0.0001-1.0062, *P_IVW_* = 0.0457). MR-Egger regression, weighted mode, and WE indicated similar results. Additionally, we discovered no causal link between PCOS and toothache (*P_IVW_* >0.05), LH and painful gums, loose teeth, or toothache (*P_IVW_* >0.05), or AMH and Estradiol level with any of the four oral diseases (*P_IVW_* >0.05).

**Conclusion:**

Our research provides new insights and references for exploring the effects of PCOS and related hormones on oral inflammatory lesions. For patients with PCOS, especially those with elevated LH levels, early intervention measures should be taken to prevent the occurrence of oral inflammatory diseases.

## 1 Introduction

Polycystic ovary syndrome (PCOS) is the primary cause of anovulatory infertility in women, affecting 5-15% of women of reproductive age globally (1). It is the most common endocrine disorder in this demographic. PCOS is highly heterogeneous, characterized mainly by hormonal imbalances, polycystic ovarian changes, infrequent menstruation, insulin resistance, chronic inflammation, etc (2–4). Anti-Müllerian Hormone (AMH)、Estradiol and Luteinizing Hormone (LH) are vital sex hormones in women of reproductive age, with AMH levels in PCOS patients typically 2-4 times higher than in healthy women (5). Presently, the occurrence of PCOS is thought to be associated with genetics, environment, oxidative stress, inflammatory response, gut microbiome, and autophagy among other factors (6–8). Still, the specific cause remains unclear.

Oral health is closely related to various systemic diseases, and poor oral health often reflects the overall health status of an individual (9–15). Chronic inflammation can cause damage to oral mucosa and periodontal tissues, leading to mouth ulcers and recurring inflammation in teeth supporting structures, resulting in symptoms like painful gums and loose teeth, and eventually tooth loss. Mouth ulcers and periodontitis are the most common oral inflammatory diseases, posing significant challenges to public health (16). Their etiology may be associated with oral microbial dysbiosis, host susceptibility, endocrine, and metabolic disorders (17, 18).

The relationship between PCOS and sex hormones with common oral diseases is receiving increasing attention (19) and becoming a significant focus in reproductive endocrinology (20, 21). Research has proven a close relationship between PCOS and periodontal inflammation (20, 22). Some studies have found higher probing depths in the gums of PCOS patients, suggesting an increased risk of periodontal disease (23). Meta-analyses and retrospective cohort studies further indicate a significantly increased prevalence of periodontal disease in PCOS patients (24, 25). In a study by Mutlak et al. (26), it was discovered that patients with PCOS suffering from gingivitis had higher concentrations of AMH. They recommended that all doctors refer PCOS patients to dental clinics for comprehensive oral assessment and treatment.

Past studies on their relationship mainly centered on observational research and meta-analyses, methods prone to confounding factors, and cannot exclude the possibility of reverse causality. Randomized controlled trials are considered the gold standard for etiological research but can be influenced by various experimental conditions, making implementation challenging. Mendelian randomization analysis helps overcome some deficiencies of traditional observational research methods. Since gamete occurrence and zygote formation follow Mendelian genetic laws and are irreversible, MR can effectively avoid reverse causality and confounding factors. Parental genetic variations are randomly distributed to offspring, so MR analysis is somewhat equivalent to conducting a randomized controlled experiment.

In this study, we utilized large sample GWAS data and bidirectional MR methods to infer the causal relationships between PCOS, three related hormones, and common oral inflammatory diseases.

## 2 Materials and methods

### 2.1 Study design

**Figure 1:**
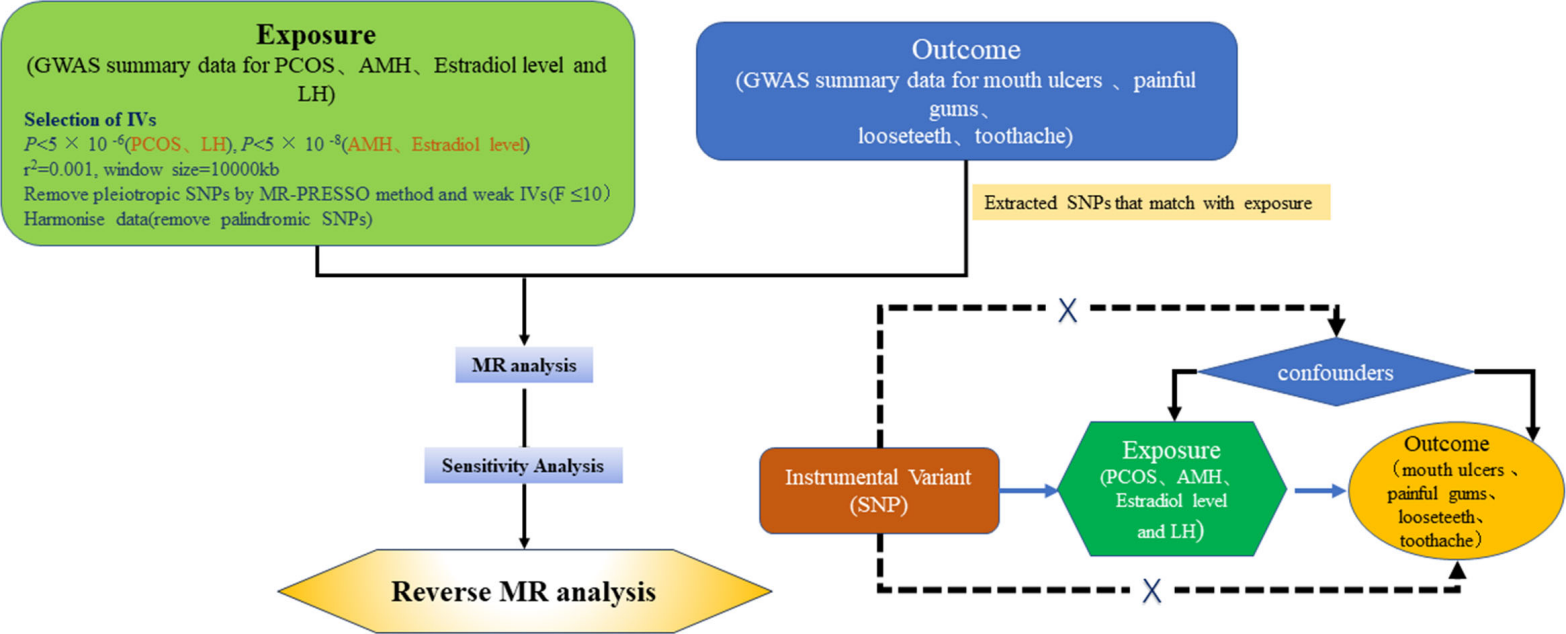
Overview of MR analyses process.

Our research project adopted a bidirectional Mendelian randomization approach, designating PCOS and three sex hormones as exposure variables and oral lesions as outcomes, to analyze the causal relationship between PCOS, sex hormones, and four types of oral lesions. Subsequently, we used the four types of oral lesions as exposures and PCOS with three sex hormones as outcomes to reverse analyze the causal relationships (See **Figure 1**).

### 2.2 Data sources

Summary data for GWAS of PCOS and four types of oral inflammatory diseases were obtained from the Finnish database and included only European populations. The PCOS sample consisted of 118,870 individuals, with 642 cases and 118,228 controls, containing a total of 16,379,676 SNPs. The mouth ulcers sample totaled 336,138 cases, with 10,894,596 SNPs. The samples for painful gums, loose teeth, and toothache totaled 461,113 cases each, containing 9,851,867 SNPs. AMH summary data were sourced from a study by Verdiesen et al. (27), incorporating 7049 premenopausal women of European ancestry with 8,298,138 SNPs. Estradiol and LH summary data were sourced from research by Ruth et al. (28) and Sun et al. (29), containing 206,927 and 3,301 European samples, respectively, with corresponding SNP totals of 16,136,413 and 10,534,735.

### 2.3 Selection of instrumental variables

The SNPs chosen as instrumental variables needed to fulfill three assumptions of Mendelian randomization: a correlation with exposure; independence, free from other confounding influences; and an effect on the outcome only through exposure. We set a threshold of *P*< 5 × 10^–6^ for SNP selection, simultaneously calculating the F-value to test the strength of IV, and choosing strongly correlated instrumental variables with F >10 (30). The linkage disequilibrium coefficient was set to r^2^<0.001, with R^2^-values for linkage disequilibrium distance at 10000 kb (31). Finally, we conducted multiple sensitivity analyses, including MR-Egger regression, to exclude pleiotropy interference and manually removed SNPs associated with confounding factors.

### 2.4 Statistical analysis

We employed four methods for two-sample MR analysis, with inverse variance-weighted (IVW) considered the primary research method. The IVW involves calculating the Wald ratio for each SNP and then combining them using the inverse of the variance as weights (32), resulting in a more accurate overall effect estimate. In addition, MR-Egger regression (33), weighted median method, and weighted mode were used as supplementary methods to analyze exposure-outcome correlations.

Sensitivity checks were conducted to validate the robustness of MR results. We assessed heterogeneity between the two samples using Cochran’s Q test. By calculating the MR-Egger intercept’s relationship with 0, we determined whether there was horizontal pleiotropy among SNPs. If the intercept was greater than 0, horizontal pleiotropy was present, meaning outcomes would still exist without exposure interference. We used the MR-PRESSO global test to exclude IVs with horizontal pleiotropy and outliers (34). The leave-one-out method was employed, sequentially excluding each SNP and testing the remaining ones for robustness (35).

All data analyses were performed using R software 4.2.1, including the TwoSampleMR package (version 0.5.6), MR-PRESSO package, and MendelianRandomization package.

### 2.5 Reverse mendelian randomization analysis

To further explore whether there exists a reverse causal relationship between PCOS, three related sex hormones, and four types of oral lesions, we conducted a reverse Mendelian randomization analysis, using the four oral lesions as exposure variables and PCOS with three sex hormones as outcomes.

## 3 Results

### 3.1 Selection of instrumental variables

When PCOS is considered as the exposure variable, at the significance level of *P*<5 $\times 10^{-6}$ , 10 SNPs were identified as instrumental variables. When considering AMH and Estradiol as exposure variables at the *P*<5 $\times 10^{-8}$ significance level, 46 and 14 SNPs, respectively, were selected as conforming instrumental variables. As for LH, when it’s taken as an exposure variable at *P*<5 $\times 10^{-6}$ , 9 SNPs were chosen as suitable instrumental variables. All of the instrumental variables had an F-value greater than 10, thus eliminating the impact of weak instruments (See **Supplementary Table S1**).

### 3.2 Effect of PCOS on oral lesions

**Figure 2:**
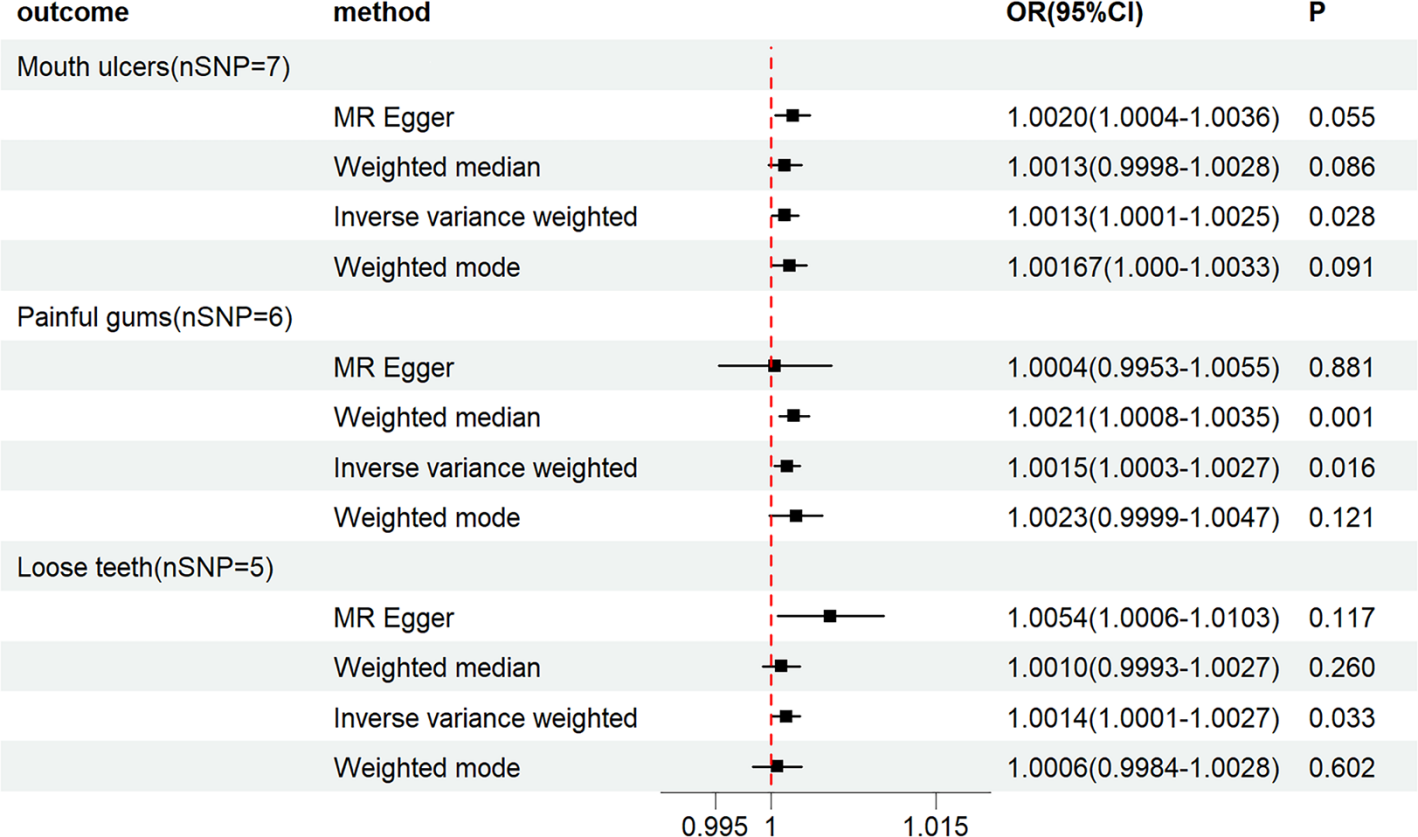
Forest plot of MR analysis results between PCOS and Mouth ulcers, painful gums, Loose teeth.

**Figure 3:**
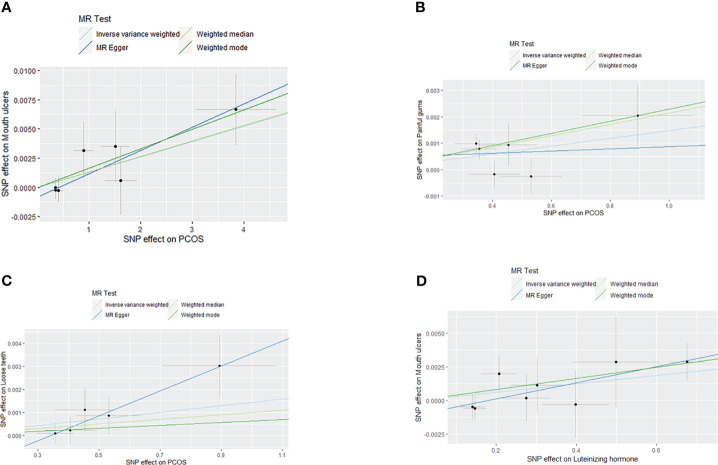
Scatter plot of MR analysis results: **(A)** scatter plot of MR analysis results between PCOS and Mouth ulcers; **(B)** scatter plot of MR analysis results between PCOS and painful gums; **(C)** scatter plot of MR analysis results between PCOS and Loose teeth; **(D)** scatter plot of MR analysis results between LH and Mouth ulcers.

Our findings reveal that PCOS is associated with an increased risk of Mouth ulcers (*OR_IVW_* = 1.0013, *95%CI:* 1.0001-1.0025, *P_IVW_* = 0.0278), painful gums (*OR_IVW_* = 1.0015, *95%CI*:1.0003-1.0027, *P_IVW_* = 0.0163), and Loose teeth (*OR_IVW_* = 1.0014, *95%CI*: 1.0001-1.0027, *P_IVW_* = 0.0328). MR-Egger regression, weighted mode, and WE indicated similar results (See **Figures 2**, **3**). Moreover, we found no causal association between PCOS and Toothache (*P_IVW_* >0.05) (See **Supplementary Table S5**).

### 3.3 Effect of PCOS-related hormones on oral lesions

**Figure 4:**
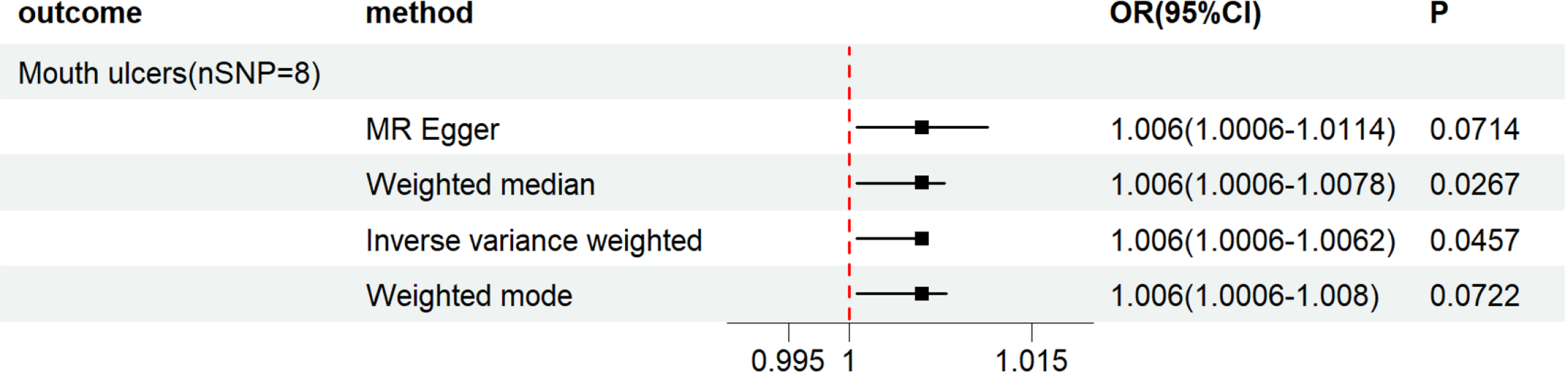
Forest plot of MR analysis results between LH and Mouth ulcers.

Our study shows that LH increases the risk of Mouth ulcers (*OR_IVW_* = 1.0031, *95%CI:* 0.0001-1.0062, *P_IVW_* = 0.0457), with MR-Egger regression, weighted mode, and WE suggesting similar outcomes (See **Figures 4**, **3**). No causal relationships were found between LH and painful gums, Loose teeth, or Toothache (*P_IVW_* >0.05) (See **Supplementary Table S8**). Simultaneously, neither AMH nor Estradiol was found to have a causal relationship with the four types of oral diseases (*P_IVW_* >0.05) (See **Supplementary Tables S6**, **S7**).

### 3.4 Sensitivity analysis

**Figure 5:**
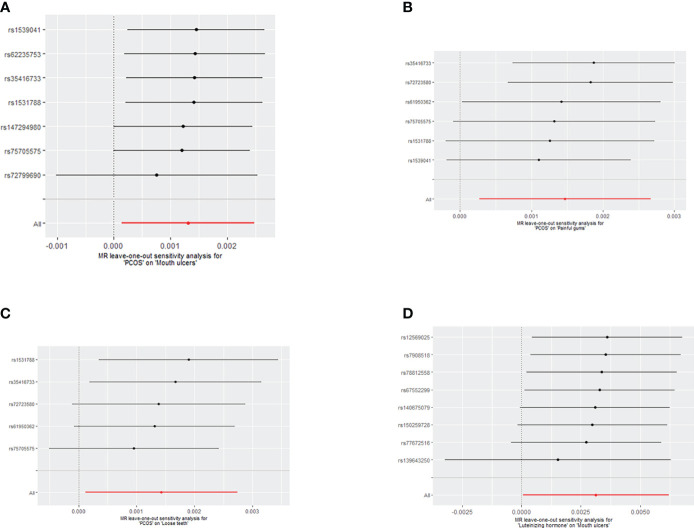
**(A)** Leave-one-out analysis of the effect of individual SNPs on the association between PCOS and the risk of Mouth ulcers; **(B)** Leave-one-out analysis of the effect of individual SNPs on the association between PCOS and the risk of painful gums; **(C)** Leave-one-out analysis of the effect of individual SNPs on the association between PCOS and the risk of Loose teeth; **(D)** Leave-one-out analysis of the effect of individual SNPs on the association between LH and the risk of Mouth ulcers.

Initially, heterogeneity tests were performed on the IV using IVW and MR-Egger methods, revealing no heterogeneity (*P*>0.05) (See **Supplementary Table S2**). The MR-Egger regression’s intercept test and MR-PRESSO global test (*P*>0.05) indicated that our selected instrumental variables did not exhibit horizontal pleiotropy, thereby excluding the influence of confounding factors (See **Supplementary Tables S3**, **S4**). The Leave-one-out test indicated that the MR results were robust (See **Figure 5**).

### 3.5 Bidirectional mendelian randomization analysis

To validate whether a bidirectional causal relationship exists between PCOS and the related three hormones and oral lesions, we considered Mouth ulcers, painful gums, Loose teeth, and Toothache as exposure variables and PCOS, AMH、Estradiol and LH as outcomes. We extracted instrumental variables for two-sample MR analysis at the significance level of *P*<5 $\times 10^{-6}$ . The results from all four MR analysis methods found no significant causal effects (*P*>0.05), indicating that there is no reverse causal relationship between PCOS and the three related hormones (AMH, Estradiol, and LH) and the four oral lesions (See **Supplementary Table S9**).

## 4 Discussion

This research is the first to utilize Mendelian randomization methods to investigate PCOS and associated hormones (AMH, Estradiol and LH) with four oral inflammatory lesions (mouth ulcers, painful gums, loose teeth, and toothache). Four types of MR analysis methods were applied for bidirectional MR analysis. The results indicate a positive correlation between PCOS and three types of oral lesions (mouth ulcers, painful gums, loose teeth), and a positive correlation between LH and the risk of oral ulcers.

In the selection of exposure variables for our study, we chose LH, E2 and AMH due to their direct involvement in ovarian function and their established roles in the pathophysiology of PCOS. While testosterone and other androgens, along with insulin levels, are indeed central to many of the disease’s consequences, our focus on LH, E2, and AMH was guided by their critical roles in ovarian follicular development and their utility as markers for ovarian reserve and function. LH and E2 are pivotal in the regulation of the menstrual cycle and are often dysregulated in PCOS, providing insight into the anovulatory aspect of the syndrome. AMH, which is typically elevated in PCOS, serves as an indicator of the granulosa cell mass and is reflective of the increased follicular count characteristic of the condition. Our aim was to elucidate aspects of PCOS pathophysiology that are directly related to ovarian dysfunction, and these hormones provide a clear window into these mechanisms.

Painful gums, loose teeth, and toothache are primary symptoms of periodontitis, resulting from toxins secreted by bacteria within dental plaque on the gums and nearby surfaces, eventually leading to tooth loss (36). Studies have reported that, compared to a healthy population, the plaque index in the oral cavity of PCOS patients is higher (37), with an increased susceptibility to soft tissue infections and worse periodontal conditions (38–40). This leads to a close association between PCOS and mouth ulcers, as well as inflammatory periodontal diseases. Further research has revealed that the risk of moderate periodontitis in untreated PCOS patients (*OR* = 5.64, *95% CI*: 2.09-15.24) was reduced to one-fifth (*OR* = 2.88, *95% CI*: 1.18-6.98) in those treated with contraceptives and metformin (41).

In light of previous research, we recognize that the relationship between PCOS and periodontal inflammation has been widely discussed, but no study has identified the specific connection between PCOS and the symptoms of inflammatory periodontal disease. Also, the association between PCOS and other inflammatory oral conditions, such as mucosal lesions, remains unreported. Past studies on PCOS and oral inflammatory diseases were primarily observational, involving only one instance of genetic variation research. In 2021, Wu et al. (42) conducted a bidirectional MR analysis on the causal connection between PCOS and periodontitis in the European population, finding no evidence of causality. Our study builds on this, further examining the relationship between PCOS and three typical symptoms of periodontitis, exploring the causal relationship between PCOS and oral inflammatory diseases.

The mechanisms through which PCOS leads to oral inflammatory diseases may involve changes in oral microbiota, oxidative stress (22, 43), inflammatory responses, bone metabolism abnormalities, and associations with hormonal imbalances and insulin resistance within PCOS patients (44). Notably, the quantity of *Capnocytophaga gingivalis* in the subgingival microbial community shows a positive correlation with estrogen levels and is closely related to PCOS oral inflammation (45). Researchers have also discovered significant reductions in *actinomycetes* and increased *spirochete* quantities in the oral microbiome of PCOS patients, which could destabilize the oral microecology and cause chronic inflammation (46–49). *Actinomycetes* are a vital group of microorganisms that maintain the stability of the oral microecosystem (50). A reduction in their quantity can disrupt this delicate microecological balance, leading to an increased migration of immune cells from the gingival sulcus. This, in turn, further activates the body’s immune system, thereby triggering the onset of persistent chronic inflammation.Furthermore, the reduction in estrogen levels in PCOS patients can increase pro-inflammatory factors, thereby enhancing the risk of oral inflammatory diseases (51). PCOS can also affect bone metabolism and absorption, leading to loose teeth and eventual tooth loss, possibly related to reduced vitamin D levels. Additionally, insulin resistance, a common comorbidity in PCOS, can induce RANKL expression, increasing bone absorption (52).

This study, utilizing bidirectional MR analysis, can eliminate confounding factors and reverse causation interference, yet some limitations remain. Firstly, our research focuses on the European population, possibly leading to certain biases. Secondly, the biological significance of SNPs as instrumental variables (IVs) is complex, and relying solely on genome-wide significance cannot ensure the validity of genetic variations. Lastly, the classification of original data precluded further subtype analysis based on ICD standards, allowing only a holistic analysis of PCOS. While the results of reverse MR analysis were not significant, they still require cautious interpretation, and future validation with a larger sample size is necessary.

## 5 Conclusion

Our bidirectional MR study indicates a potential association between PCOS and certain oral inflammatory diseases, including mouth ulcers, painful gums, and loose teeth. Notably, the findings, particularly those derived from the IVW method, warrant cautious interpretation due to the observed variability in statistical significance across different methods and the proximity of odds ratios to unity. While our study adds to the growing body of evidence on this subject, the relatively modest effect sizes suggest that these associations, although statistically significant, may have limited clinical impact. Consequently, our results should be viewed as preliminary until further corroborated by additional research. The study underscores the importance of considering a broad range of methodological approaches and highlights the need for continued investigation into the complex interplay between PCOS and oral health.

## Data availability statement

The original contributions presented in the study are included in the article/**Supplementary Material**. Further inquiries can be directed to the corresponding author.

## Author contributions

QM: Conceptualization, Data curation, Formal analysis, Investigation, Methodology, Software, Visualization, Writing – original draft, Writing – review & editing. YC: Formal analysis, Investigation, Supervision, Writing – review & editing. HG: Data curation, Funding acquisition, Project administration, Writing – review & editing. QG: Formal analysis, Investigation, Methodology, Writing – original draft. XZ: Formal analysis, Investigation, Methodology, Writing – original draft. MX: Conceptualization, Funding acquisition, Project administration, Resources, Supervision, Writing – review & editing.
